# The Effects of Vitamin D Supplementation on Signaling Pathway of Inflammation and Oxidative Stress in Diabetic Hemodialysis: A Randomized, Double-Blind, Placebo-Controlled Trial

**DOI:** 10.3389/fphar.2018.00050

**Published:** 2018-02-02

**Authors:** Hamed Haddad Kashani, Elahe Seyed Hosseini, Hossein Nikzad, Alireza Soleimani, Maryam Soleimani, Mohammad Reza Tamadon, Fariba Keneshlou, Zatollah Asemi

**Affiliations:** ^1^Anatomical Sciences Research Center, Kashan University of Medical Sciences, Kashan, Iran; ^2^Department of Internal Medicine, Kashan University of Medical Sciences, Kashan, Iran; ^3^Research Center for Biochemistry and Nutrition in Metabolic Diseases, Kashan University of Medical Sciences, Kashan, Iran; ^4^Department of Internal Medicine, Semnan University of Medical Sciences, Semnan, Iran; ^5^Department of Urology, School of Medicine, Alborz University of Medical Sciences, Karaj, Iran

**Keywords:** vitamin D supplementation, hemodialysis, signaling pathway, inflammation, oxidative stress

## Abstract

**Objective:** This study was carried out to determine the effects of vitamin D supplementation on signaling pathway of inflammation and oxidative stress in diabetic hemodialysis (HD) patients.

**Methods:** This randomized double-blind placebo-controlled clinical trial was conducted among 60 diabetic HD patients. Subjects were randomly allocated into two groups to intake either vitamin D supplements at a dosage of 50,000 IU (*n* = 30) or placebo (*n* = 30) every 2 weeks for 12 weeks. Gene expression of inflammatory cytokines and biomarkers of oxidative stress were assessed in peripheral blood mononuclear cells (PBMCs) of diabetic HD patients with RT-PCR method.

**Results:** Results of RT-PCR indicated that after the 12-week intervention, compared to the placebo, vitamin D supplementation downregulated gene expression of interleukin (IL)-1β (*P* = 0.02), tumor necrosis factor alpha (TNF-α) (*P* = 0.02) and interferon gamma (IFN-γ) (*P* = 0.03) in PBMCs of diabetic HD patients. Additionally, vitamin D supplementation, compared to the placebo, downregulated gene expression of transforming growth factor beta (TGF-β) (*P* = 0.04), protein kinase C (PKC) (*P* = 0.001), and mitogen-activated protein kinases 1 (MAPK1) (*P* = 0.02) in PBMCs of diabetic HD patients. Although not significant, vitamin D supplementation let to a reduction of nuclear factor kappa B (NF-kB) (*p* = 0.75) expression in PBMCs isolated from diabetic patients compared to the placebo group. There was no statistically significant change following supplementation with vitamin D on gene expression of interleukin (IL)-4, IL-6, and vascular endothelial growth factor (VEGF) in PBMCs of diabetic HD patients.

**Conclusions:** Overall, we found that vitamin D supplementation for 12 weeks among diabetic HD patients had beneficial effects on few gene expression related to inflammation and oxidative stress.

**Clinical trial registration:** IRCT201701035623N101. Registered on January 8, 2017.

## Introduction

Increased inflammatory cytokines as a necessary part of chronic kidney disease (CKD) has been identified and associated with cardiovascular disease (CVD), protein-energy malnutrition, all-cause mortality, and decreased glomerular filtration rate (GFR) (Stenvinkel et al., 1999; Akchurin and Kaskel, 2015). Increased biomarkers of inflammation, such as interleukin-1beta (IL-1β) and tumor necrosis factor alpha (TNF-α) were negatively linked to the measures of kidney action and directly with albuminuria (Gupta et al., 2012). Furthermore, various factors, including increased oxygen metabolism, uremic toxicity, increased inflammatory factors, lack of antioxidant vitamins and microelements, and dialysis procedure in patients with CKD would result in oxidative stress (Stepniewska et al., 2015).

Prior studies have documented that hypovitaminosis D is very prevalent in individuals with CKD (de Boer, 2008; Cupisti et al., 2015). In a study by Bhan et al. (2010) hypovitaminosis D was reported 50–90% in dialysis subjects. On the other hand, beneficial effects of vitamin D on biochemical variables have reported in hemodialysis (HD) patients (Ibrahim et al., 2015), subjects with type 2 diabetes mellitus (T2DM) (Calvo-Romero and Ramiro-Lozano, 2016) and polycystic ovary syndrome (Maktabi et al., 2017). Few studies have evaluated the effects of vitamin D on gene expression related to inflammation and oxidative stress. Choi et al. (2013) shown significant reductions in gene expression of interleukin 6 (IL-6) and tumor necrosis factor alpha (TNF-α) in vitamin D-treated rats. In addition, *in vitro* studies have shown that the release of TNF-α can be inhibited by calcitriol in a dose-dependent fashion (Müller et al., 1992). Another study indicated that vitamin D through vitamin D receptor (VDR) suppressed TNF-α-induced nuclear factor kappa B (NF-κB) activation and IL-6 up-regulation in epithelial cell isolated from transgenic mice and colitis animal model (Liu et al., 2013). However, another study supported no significant changes in TNF-α and IL-6 levels in high-dose vitamin D administration in healthy overweight individuals following a 12-week progressive resistance exercise training program (Carrillo et al., 2012).

The molecular mechanism of vitamin D in modulating inflammatory cytokines and oxidative stress are still unclear. Mitogen-activated protein kinases (MAPKs) participate in signaling mechanisms in responses to pro-inflammatory factors as well as NF-κB are thought to play an important function in the regulation of pro-inflammatory cytokines in cellular responses (Baldassare et al., 1999). To our knowledge, data on the effects of vitamin D supplementation on signaling pathway of inflammation and oxidative stress in diabetic HD patients are limited and controversial. The purpose of this study was, therefore, conducted to evaluate the effects of vitamin D supplementation on signaling pathway of inflammation and oxidative stress in diabetic HD patients.

## Methods

### Trial design and participants

This randomized double-blind placebo-controlled clinical trial, registered in the Iranian website for registration of clinical trials as http://www.irct.ir:IRCT201701035623N101, was carried out among 60 diabetic HD subjects aged 18-80 years who were referred to the Akhavan Clinic in Kashan, Iran, from January 2017 to April 2017. This research was carried out in accordance with the Declaration of Helsinki and the Research Ethics Committee of Kashan University of Medical Sciences (KAUMS) approved the study protocol, and informed consent was taken from all subjects. The main exclusion criteria from the study were as follows: taking vitamin D, antioxidant and/or anti-inflammatory supplements such as vitamins E and C, omega-3 fatty acids, and taking immunosuppressive medications within 3 months prior to the enrollment in the study.

### Study design

At first, all patients were randomized into two groups to receive either 50,000 IU of vitamin D or placebo (*n* = 30 in each group) every 2 weeks for 12 weeks. Vitamin D supplements and placebos were produced by Zahravi Pharmaceutical Company, Tabriz, Iran, and Barij Essence Pharmaceutical Company, Kashan, Iran, respectively. In the current study, we used the above-mentioned dose of vitamin D based on the tolerable upper intake level (4,000 IU/day) of vitamin D (Ross et al., 2011), as well as dosage recommended in previous studies among HD patients (Armas et al., 2013; Mose et al., 2014). Compliance to the vitamin D consumption was evaluated through determination of serum 25 (OH) vitamin D values and asking subjects to return the medication containers. Both dietary 3-day food records [analyzed by nutritionist IV software (First Databank, San Bruno, CA)] and physical activity records were taken at baseline, weeks 3, 6, 9, and 12 of the intervention.

### Assessment of anthropometric measures

Body weight and height were quantified in a fasting status using a digital scale (Seca, Hamburg, Germany) at baseline and after the 12-week intervention. Body mass index (BMI) was calculated by weight and height measurements [weight (kg)/height (m^2^)].

### Assessment of outcomes

Primary outcomes were inflammatory cytokines expression and secondary outcomes were biomarkers of oxidative stress expression. We selected the following inflammatory cytokines and proteins [IL-1β, IL-4, IL-6,TNF-α, interferon gamma (IFN-γ), transforming growth factor beta (TGF-β), protein kinase C (PKC), MAPK1, vascular endothelial growth factor (VEGF) and NF-κB] as they play a very important role in signaling pathways of oxidative stress (Geraldes and King, 2010; Baker et al., 2011; Tanti et al., 2012; Chen et al., 2015).

### Isolation of peripheral blood mononuclear cells (PBMCs)

At baseline and endpoint of the intervention, 15 mL samples of fasting blood were taken at reference laboratory of KAUMS, Kashan, Iran. PBMCs were isolated from blood by centrifugation through a Ficoll-Hypaque (Denholm and Wolber, 1991) followed by Percoll. In summary, 10 mL of blood with an equal volume of phosphate-buffered saline (PBS) was mixed (Dagur and McCoy, 2015). Layer diluted blood over Ficoll-Hypaque solution, using 3 parts diluted blood to 2 parts Ficoll-Hypaque and centrifuged 30 min at 500 × g, 25°C. Then, using a sterile Pasteur pipet, carefully collected PBMCs, and located at the interface between the plasma (upper layer) and Ficoll-Hypaque solution (Dagur and McCoy, 2015). Cells to a 50 mL centrifuge tube were transferred, then after adding 10 mL PBS, were centrifuged 10 min at 400 × g, 4°C. The supernatant was discarded and was resuspended cells in 4 mL PBS. Following previous step, 1.65 mL of 10× Hanks balanced salt solution with 10 mL Percoll was mixed. Eight mL Percoll solution with 4 mL mononuclear cells in a silanized 10 × 1.5 cm round-bottom polypropylene tube was mixed (Dagur and McCoy, 2015). Mixing thoroughly by inverting tube three or four times was done. Then, 25 min at 370 × g, 25°C was centrifuged. PBMCs were aspirated by gentle pipetting into a clean test tube. After centrifugation, monocytes appear as a cloudy layer in the top 5 mm of the gradient (Dagur and McCoy, 2015).

### RNA extraction and real-time PCR

To RNA extraction, we used the RNX-plus kit (Cinnacolon, Tehran, Iran). RNA were extracted just after lymphocyte extraction from fresh 5 mL peripheral blood and less than 1 million cells was used for RNA extraction with the trizol reagent (Invitrogen, USA). Following extraction of the total RNAs from each sample, RNA quantification were performed by UV spectrophotometer. Each samples OD 260/280 ratio between 1.7 and 2.1 was intended showing no contamination with both protein and DNA (Dunkley et al., 2008). The isolated RNA was reverse transcribed to cDNA library using moloney murine leukemia virus reverse transcriptase. Gene expression of IL-1β, IL-4, IL-6,TNF-α, IFN-γ, TGF-β, PKC, MAPK1, VEGF, and NF-κB were evaluated by quantitative RT-PCR, using the LightCycler technology (Roche Diagnostics, Rotkreuz, Switzerland) with SYBR green detection and Amplicon Kit (Table 1). Glyceraldehyde-3-phosphate dehydrogenase primers were used as housekeeping gene. To design primers, Primer Express Software (Applied Biosystems, Foster City) and Beacon designer software (Takaposizt, Tehran, Iran) were used. Relative transcription levels were calculated by the method of Pffafi or 2^−ΔΔCT^. In the current study, we quantified gene expression levels related to inflammation and oxidative stress in PBMCs from diabetic HD patients. PBMCs from venous blood samples are the most available tissue for analysis of gene expression (Mizuarai et al., 2010). In addition, gene expression levels related to inflammation and oxidative stress in PBMCs are more accurate and may provide more valuable information than plasma concentrations (Mizuarai et al., 2010). Few studies have previously assessed gene expression levels of inflammation and oxidative stress in PBMCs from diabetic patients (Xavier et al., 2015; Mazloom et al., 2016).

**Table 1:** Specific primers used for real-time quantitative PCR.

**Gene**	**Primer**	**Product size (bp)**	**Annealing temperature (C)**
GAPDH	F: GAGTCAACGGATTTGGTCGT	223	58
	R: TTGATTTTGGAGGGATCTCG		
IL1-β	F: GATGGCTTATTACAGTGGCAATG	137	59
	R: AGTGGTGGTCGGAGATTCG		
IL-4	F: CTCACAGAGCAGAAGAACAC	221	60
	R: TGGTTGGCTTCCTTCACAG		
IL-6	F: GTAGTGAGGAACAAGCCAGAG	286	60
	R: TGACCAGAAGAAGGAATGCC		
TNF-α	F: GAGCCAGCTCCCTCTATTTATG	187	60
	R: CTACATGGGAACAGCCTATTGT		
IFN-γ	F: GGTTCTCTTGGCTGTTACTG	250	59.5
	R: TGTCTTCCTTGATGGTCTCC		
VEGF	F: TGCAGATTATGCGGATCAAACC	150	58.5
	R:TGCATTCACATTTGTTGTGCTGTAG		
NFK-β	F: GCTGAGTCCTGCTCCTTC	202	58
	R: GTCTATTTGCTGCCTTGTGG		
TGF-β	F: ACTACTACGCCAAGGAGGTC	250	59
	R: CGGTTGCTGAGGTATCGC		
PKC	F: CGTCCTCATTGTCCTCGTAAG	249	59
	R: TCATTCCTGCTGGTCAAATCC		
MAPK1	F: GGAACAGCACCTCCACTATTT	226	60
	R: GCCACAATGTCTGCGTATCT		

*GAPDH, glyceraldehyde-3-Phosphate dehydrogenase; IL-1β, interleukin-1β; IFN-γ, interferon gamma; MAPK1, mitogen-activated protein kinases 1; NF-κB, nuclear factor kappa B; PBMCs, peripheral blood mononuclear cells; PKC, protein kinase C; TNF-α, tumor necrosis factor alpha; TGF-β, transforming growth factor beta; VEGF, vascular endothelial growth factor*.

### Randomization

Randomization assignment was carried out using computer-generated random numbers. Randomization and allocation concealment were conducted by the researchers and participants and were carried out by a trained staff at the clinic.

### Statistical methods

The Kolmogorov-Smirnov test was applied to control the normal distribution of variables. Independent sample *t*-test was used to establish changes in anthropometric measures and dietary intakes between the two groups. To determine the effects of vitamin D administration on inflammation and oxidative stress expression, we used independent sample *t*-test. *P* < 0.05 were considered statistically significant. All statistical analyses conducted using the Statistical Package for Social Science version 18 (SPSS Inc., Chicago, Illinois, USA).

## Results

**Figure 1:**
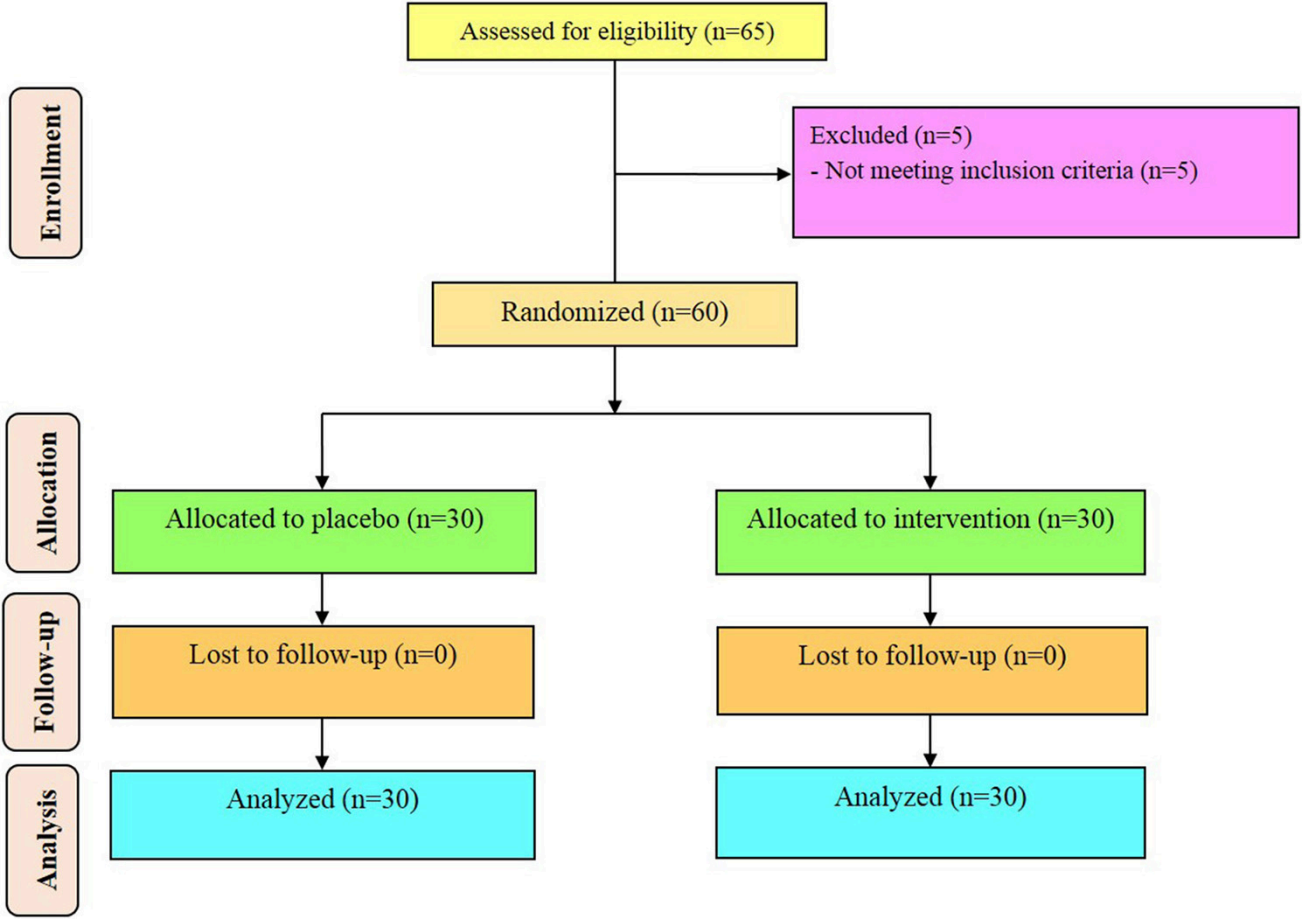
Summary of patient flow diagram.

Sixty diabetic HD patients [vitamin D and placebo (*n* = 30 each group)] completed the trial (Figure 1). On average, the rate of compliance in our study was high, such that more than 100% of capsules were taken throughout the study in both groups. No side effects were reported following supplementation of vitamin D in diabetic HD patients throughout the study. Potential side effects of vitamin D supplementation monitored by evaluating the serum levels of calcium every month, as well as, monitoring clinical symptoms among participants including, anorexia, nausea, and vomiting.

Distribution of gender, participants' mean age, height, baseline weight and BMI as well as their change, and years of dialysis of study participants were not statistically different between the two groups (Table 2).

**Table 2:** General characteristics of study participants.

	**Placebo group (*n* = 30)**	**Vitamin D group (*n* = 30)**	***P*^a^**
**GENDER (%)**			
Male	20 (66.7)	19 (63.4)	0.78^†^
Female	10 (33.3)	11 (36.6)	
**TYPE OF DIABETES (%)**			
Type 1	2 (6.7)	2 (6.7)	1.00^†^
Type 2	28 (93.3)	28 (93.3)	
Smoking (%)	3 (10.0)	3 (10.0)	1.00^†^
Duration of DM (year)	18.9 ± 6.0	19.1 ± 5.4	0.87
Insulin therapy (%)	30 (100.0)	30 (100.0)	1.00^†^
Age (y)	60.7 ± 14.3	57.1 ± 13.4	0.31
Height (cm)	162.5 ± 8.0	163.5 ± 8.1	0.65
Weight at study baseline (kg)	71.2 ± 15.7	69.5 ± 11.8	0.65
Weight at end-of-trial (kg)	71.2 ± 15.7	70.1 ± 11.9	0.75
Weight change (kg)	0.008 ± 1.3	0.5 ± 1.3	0.12
BMI at study baseline (kg/m^2^)	27.0 ± 6.0	26.1 ± 4.5	0.51
BMI at end-of-trial (kg/m^2^)	27.0 ± 5.9	26.3 ± 4.5	0.61
BMI change (kg/m^2^)	0.002 ± 0.5	0.2 ± 0.5	0.11
Years on dialysis	3.7 ± 1.0	3.9 ± 1.2	0.56
Cancer (%)	2 (6.7)	2 (6.7)	1.00^†^
CAD (%)	22 (73.3)	21 (70.0)	0.77^†^
CVD (%)	5 (16.7)	6 (20.0)	0.73^†^
Hypertension (%)	28 (93.3)	29 (96.7)	0.55^†^

*Data are means ± SDs*.

a *Obtained from independent t-test*.

† *Obtained from Pearson Chi-square test*.

*CAD, coronary artery disease; CVD, cerebrovascular disease; DM, diabetes mellitus*.

Considering the 3-day dietary records obtained during the intervention, there was no significant difference in terms of dietary macro- and micro-nutrient intakes between vitamin D and placebo groups (Data not shown).

**Figure 2:**
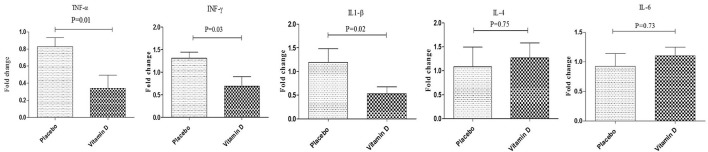
Effect of 12-week supplementation with vitamin D or placebo on expression ratio of TNF-α, IFN-γ, IL-1β, IL-4, and IL-6 genes in PBMCs of diabetic HD subjects. *P*-value was obtained from independent sample *t*-test. Data are means ± standard deviation.

After 12 weeks of intervention, compared to the placebo, vitamin D supplementation significantly increased serum 25-OH-vitamin D (+11.1 ± 4.2 vs. +0.5 ± 3.9 ng/mL, *P* < 0.001). Results of RT-PCR indicated that compared to the placebo, vitamin D supplementation downregulated gene expression of IL-1β (*P* = 0.02), TNF-α (*P* = 0.02), and IFN-γ (*P* = 0.03) in PBMCs of diabetic HD patients (Figure 2). There was no statistically significant change following supplementation with vitamin D on gene expression of IL-4 and IL-6 in PBMCs of diabetic HD patients.

**Figure 3:**
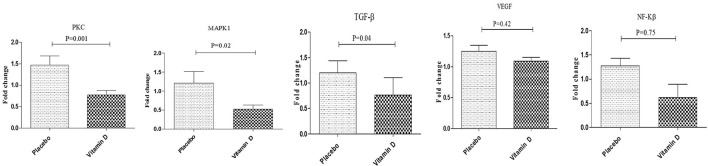
Effect of 12-week supplementation with vitamin D or placebo on expression ratio of PKC, MAPK1, TGF-β, NF-κB, and VEGF genes in PBMCs of diabetic HD subjects. *P*-value was obtained from independent sample *t*-test. Data are means ± standard deviation.

Additionally, vitamin D supplementation, compared to the placebo, downregulated gene expression of TGF-β (*P* = 0.04), PKC (*P* = 0.001), and MAPK1 (*P* = 0.02) in PBMCs of diabetic HD patients (Figure 3). Although not significant, vitamin D supplementation let to a reduction of NF-kB (*p* = 0.75) expression in PBMCs isolated from diabetic patients compared to the placebo group. We did not observe any significant change following supplementation with vitamin D on gene expression of VEGF in PBMCs of diabetic HD patients.

## Discussion

We found that vitamin D supplementation for 12 weeks among diabetic HD patients had beneficial effects on few gene expression related to inflammation and oxidative stress. To our knowledge, this is the first report of the effects of vitamin D supplementation on gene expression related to inflammation and oxidative stress in diabetic HD patients.

HD subjects are susceptible to several metabolic complications, including increased inflammation and oxidative stress (Kotur-Stevuljevic et al., 2012). Our data demonstrated that vitamin D supplementation for 12 weeks to diabetic HD subjects downregulated gene expression of IL-1β, TNF-α, and IFN-γ compared to the placebo, but did not affect gene expression of IL-4 and IL-6. A study in healthy endurance-trained runners has demonstrated the inverse relationship between vitamin D and TNF-α levels (Willis et al., 2012). Furthermore, in line with our findings, 50 mug/day of vitamin D supplementation for 9 months was able to suppress the production of TNF-α in subjects with congestive heart failure (Schleithoff et al., 2006). 1,25-(OH)2D3 dose-dependently also suppressed the production of IL-α, IL-6, and TNF-α by Escherichia coli lipopolysaccharide-stimulated monocytes (Müller et al., 1992). However, one study has documented no significant changes in TNF-α and IL-6 concentrations in high-dose vitamin D supplementation-healthy overweight populations after 12 weeks (Carrillo et al., 2012). Chronic inflammation in CKD is not only associated with macro- and micro-cardiovascular outcomes, such as atherosclerosis, but is also one of the main players in the progress of malnutrition/protein-energy wasting, which in turn resulted in the description of the malnutrition-inflammation-cachexia syndrome in CKD/ESRD patients (Kalantar-Zadeh, 2005). Hypoalbuminemia result from chronic inflammation and malnutrition is strongly related to mortality in dialysis populations (Kalantar-Zadeh et al., 2005; de Mutsert et al., 2009). Pro-inflammatory factors may directly lead to anorexia via the effect on the brain. Furthermore, inflammatory cytokines, particularly IL-6, may be related to depression in CKD/ESRD patients, which by itself is a predictor of morbidity and mortality (Taraz et al., 2015) and may result in decreasing nutrient intake. Therefore, vitamin D due to its useful effects on inflammatory cytokines may be benefit to decrease clinical and metabolic symptoms in diabetic HD patients.

This study demonstrated that vitamin D supplementation for 12 weeks downregulated gene expression of TGF-β, PKC, and MAPK1 in PBMCs of diabetic HD patients, but did not influence gene expression of VEGF and NF-Kβ. Supporting our study, vitamin D administration at a dosage of 50,000 IU/week for 8 weeks in VD-deficient subjects with PCOS significantly decreased the bioavailability of TGF-β1 (Irani et al., 2015). In addition, 1,25-(OH)2D3 decreased gene expression of MAPK phosphorylation in macrophages (Xu et al., 2016). In another study, vitamin D protected human endothelial cells from ionizing radiation induced/oxidative stress by modulating the MAPKs/SirT1 axis (Marampon et al., 2016). Doxercalciferol also significantly decreased PKCα levels suggesting that PKC-mediated cardiac hypertrophy may be related to vitamin D deficiency (Choi et al., 2011). Excessive evidence exists suggesting that MAPK signaling pathways are critical for the synthesis and amplification of inflammatory factors (Carter et al., 1999). Especially, MAPK activation was closely associated with cytokine transcription and translation, which in turn make the target of anti-inflammatory treatment (Kaminska, 2005). However, calcitriol treatment had no significant effect on gene expression of TGF-β in microcardiovascular endothelial cells (Gonzalez-Curiel et al., 2016). In addition, both the low (1 nM) and the high (100 nM) doses of vitamin D3 used *in vitro* for 48 h did not able to restore the decreased gene expression of PKC isoenzymes in the T cells of systemic lupus erythematosus subjects (Czifra et al., 2014). Oxidative stress in patients with CKD may generate by uremic toxicity, persistent inflammatory state, deficiency of vitamins and microelements, and dialysis procedure itself (Stepniewska et al., 2015). Increased biomarkers of oxidative stress and free radicals in HD subjects are correlated with increased CVD risk and a large number of diseases related to uremia (Taccone-Gallucci et al., 2010).

## Conclusions

Overall, we found that vitamin D supplementation for 12 weeks among diabetic HD patients had beneficial effects on some gene expression related to inflammation and oxidative stress. This suggests that vitamin D supplementation may confer advantageous therapeutic potential for HD patients. Further research is needed in other participants and for longer periods to determine the efficacy of vitamin D supplementation.

## Limitations

The limitations of our findings include short duration. Long-term interventions might result in better effects in other gene expression related to inflammation and oxidative stress. In addition, due to funding limitations in developing countries, we did not evaluate some gene expression related to insulin and lipid, and reactive oxygen species levels. Further investigations are required to evaluate this outcome. We did not know baseline levels of vitamin D among our participants, therefore, we were not able to comment on their commended dosage of vitamin D by the Kidney Disease Outcomes Quality Initiative (K/DOQI) guidelines. This limitation should be considered in the interpretation of our findings.

## Availability of data and materials

The primary data for this study is available from the authors on direct request.

## Author contributions

ZA contributed in conception, data collection and manuscript drafting; HH, ES, HN, AS, MS, FK, and MT contributed in conception, data collection and manuscript drafting; All authors read and approved the final version of the paper.

### Conflict of interest statement

The authors declare that the research was conducted in the absence of any commercial or financial relationships that could be construed as a potential conflict of interest.

## Abbreviations

HD: hemodialysis
PBMCs: peripheral blood mononuclear cells
IL: interleukin
TNF-α: tumor necrosis factor alpha
IFN-γ: interferon gamma
TGF-β: transforming growth factor beta
PKC: protein kinase C
MAPK1: mitogen-activated protein kinases 1
VEGF: vascular endothelial growth factor
NF-κB: nuclear factor kappa B
CVD: cardiovascular disease
CKD: chronic kidney disease
GFR: glomerular filtration rate
VDR: vitamin D receptor
BMI: Body mass index.
