# Expression of Concern: The Effects of Vitamin D Supplementation on Signaling Pathway of Inflammation and Oxidative Stress in Diabetic Hemodialysis: A Randomized, Double-Blind, Placebo-Controlled Trial

**DOI:** 10.3389/fphar.2020.602201

**Published:** 2020-09-11

**Authors:** Frontiers Editorial Office

**Affiliations:** Frontiers Media SA, Lausanne, Switzerland

**Keywords:** vitamin D supplementation, hemodialysis, signaling pathway, inflammation, oxidative stress

With this notice, Frontiers states its awareness of concerns regarding the validity of the participant data in this study. An investigation is currently being conducted by Kashan University of Medical Sciences research ethics committee. This expression of concern has been posted while Frontiers awaits the outcome of that investigation and will then be updated accordingly.

